# The Patient Repository for EEG Data + Computational Tools (PRED+CT)

**DOI:** 10.3389/fninf.2017.00067

**Published:** 2017-11-21

**Authors:** James F. Cavanagh, Arthur Napolitano, Christopher Wu, Abdullah Mueen

**Affiliations:** ^1^Department of Psychology, University of New Mexico, Albuquerque, NM, United States; ^2^Department of Computer Science, University of New Mexico, Albuquerque, NM, United States

**Keywords:** EEG, open data, pattern classification, databases as topic, clinical neuroscience

## Abstract

Electroencephalographic (EEG) recordings are thought to reflect the network-wide operations of canonical neural computations, making them a uniquely insightful measure of brain function. As evidence of these virtues, numerous candidate biomarkers of different psychiatric and neurological diseases have been advanced. Presumably, we would only need to apply powerful machine-learning methods to validate these ideas and provide novel clinical tools. Yet, the reality of this advancement is more complex: the scale of data required for robust and reliable identification of a clinical biomarker transcends the ability of any single laboratory. To surmount this logistical hurdle, collective action and transparent methods are required. Here we introduce the Patient Repository of EEG Data + Computational Tools (PRED+CT: predictsite.com). The ultimate goal of this project is to host a multitude of available tasks, patient datasets, and analytic tools, facilitating large-scale data mining. We hope that successful completion of this aim will lead to the development of novel EEG biomarkers for differentiating populations of neurological and psychiatric disorders.

## Introduction

There is a critical need to standardize and quantify the diagnostic criteria for psychiatric and neurological disorders. A lack of uniform diagnostic schema and reliance on phenotypic assessment has resulted in critical gaps in clinical practice between institutions. A growing number of reports suggest that biomarkers or endophenotypes (endogenous phenotypes) will be more effective for diagnosis and disease classification than an expanded phenotypic characterization (Gould and Gottesman, 2006; Diaz-Arrastia et al., 2009; Robbins et al., 2012).

Our long-term goal is to develop and maintain an open-source website that leverages the power of collective action to address this need using electroencephalography (EEG). By quantifying the emergence of psychological operations at their source, EEG provides a mechanistic means to transcend the descriptive, correlative, and phenotypic descriptions of symptomatology that currently characterizes this field. EEG is less expensive, more readily available, highly portable, and simpler to operate than competing imaging recourses, making it logistically viable.

While there are a large number of open-source EEG data repositories, these are sparsely populated (**Table 1**). Patient-specific online repositories tend not to have EEG data. While some sites may contain both EEG data and patient groups, they sometimes require formal requests and selective processes for data acquisition, and tend to not include matched controls. The absence of open-source software infrastructure for standardized and large-scale EEG data reflects a failure of basic biomedical planning and initiative. We aim to fill this gap with a one-stop open-source site for gathering, storing, and analyzing clinically relevant data. In this report we present the Patient Repository of EEG Data + Computational Tools (PRED+CT^1^).

**Table 1:** Examples of online EEG data repositories.

**Sparsely populated online EEG data repositories**
• http://archive.ics.uci.edu/ml/datasets/EEG+Database
• http://engineuring.wordpress.com/2009/07/08/downloadable-eeg-data/
• http://headit.ucsd.edu/
• http://openvibe.inria.fr/?q=datasets
• http://sccn.ucsd.edu/∼arno/fam2data/publicly_available_EEG_data.html
• http://sites.google.com/site/projectbci/
• http://www.bbci.de/competition/
• http://www.brainsignals.de/
• http://www.cs.colostate.edu/eeg/eegSoftware.html\#keirndata
• http://www.eecs.qmul.ac.uk/mmv/datasets/deap/
• http://www.phypa.org/benchmarking.html
• http://www.physionet.org/pn4/eegmmidb/
• http://www.physionet.org/pn6/chbmit/
• http://www2.hu-berlin.de/eyetracking-eeg/testdata.html
• http://sleeptight.isr.uc.pt/ISRUC_Sleep/
• http://www.ceams-carsm.ca/en/mass
• http://www.tcts.fpms.ac.be/~devuyst/#Databases
• see: https://sccn.ucsd.edu/∼arno/fam2data/publicly_available_EEG_data.html
**Patient-specific online repositories that may contain EEG data**
Autism:
• https://sfari.org/resources/autism-cohorts/simons-vip
• https://ndar.nih.gov/ndar_data_dictionary.html;jsessionid=7D268A92ACF3FCC2EEA35BF07892D394.node1
• http://aed.newcastle.edu.au/
Epilepsy:
• http://www.fdm.uni-freiburg.de/groups/timeseries/epi/EEGData/
• http://epileptologie-bonn.de/cms/front_content.php?idcat=193&lang=3&changelang=3
• http://epilepsy.uni-freiburg.de/freiburg-seizure-prediction-project/eeg-database
• http://ntsa.upf.edu/downloads/andrzejak-rg-schindler-k-rummel-c-2012-nonrandomness-nonlinear-dependence-and
Epilepsy (intracranial recordings):
• https://www.ieeg.org/
Traumatic brain injury:
• https://fitbir.nih.gov/
Alzheimer’s disease:
• http://adni.loni.usc.edu/
**Multi-purpose data repositories with restrictive access**
• http://www.brainnet.net/
• https://physionet.org/physiobank/database/#neuro


*Note that this is not an exhaustive list of EEG data sharing sites. PRED+CT would be a beneficial addition to this ecosystem due to its clear focus on pattern classification of patient EEG. Moreover, PRED+CT could link to external sites, repositories, and databases beneficial to this aim, facilitating an open network of interactive sites.*

## Current and Future Use of EEG As A Biomarker

Electroencephalography-based biomarkers are particularly salient due to current widespread use in neurology clinics, which increases the likelihood that a novel advancement will have immediate clinical significance. Some neural deficits like epilepsy are objectively diagnosable following an EEG; other complications like tumors and stroke can be inferred. Yet disorders that affect higher cognitive functions remain opaque following any type of routine imaging. The future use of EEG as a clinical biomarker aims to capitalize on this existing diagnostic infrastructure via knowledge advancements, facilitating greater diagnostic utility from already routine scanning sessions.

Electroencephalography is uniquely sensitive to canonical neural operations which underlie emergent psychological constructs (Fries, 2009; Siegel et al., 2012; Cavanagh and Castellanos, 2016), making it well suited for discovery of aberrant neural mechanisms that underlie complicated disease states (Insel et al., 2010; Montague et al., 2012). As an example, consider how error-related EEG activities can sensitively and specifically dissociate generalized anxiety participants from healthy controls (Cavanagh et al., 2017). This finding follows positive reports from two meta-analyses with 37 and 46 studies, and 1757 and 1616 participants, respectively (Moser et al., 2013; Cavanagh and Shackman, 2014). If even a fraction of the studies included in these meta-analyses were openly available, a widely generalizable set of discriminating features would already be available for extended research and possible clinical application.

## The Need for An Online Repository Dedicated to this Purpose

Findings in cognitive neuroscience tend to advance through independent laboratories working separately, with each group developing their own stimulus presentation tasks and data analysis parameters. While this flexibility is beneficial, it is also a threat to external validity. Generalizability is critical to consider since the scale of data required to effectively characterize clinical biomarkers transcends the abilities of any single laboratory. To realize this goal of differential diagnostic biomarkers, open-source collaboration across institutions will be required. Currently, the field of EEG lacks even a rudimentary foundation for this goal.

The need for open-source data sharing and a focus on replicability has been widely approached in the MRI community, with varied types of data repositories (Gorgolewski et al., 2015; Eickhoff et al., 2016; Iyengar, 2016; Nichols et al., 2017; Poldrack et al., 2017), including patient-specific databases and depictions of classification goals (Woo et al., 2017). PRED+CT uses the OpenfMRI project (Poldrack et al., 2013) as a model. PRED+CT will not only be the first open-source EEG database for patient data, but it will work to standardize assessment and analytic tools, facilitating the overarching goal of distributed data collection and data mining.

## Prior Approaches

It is important to note that although this basic clinical goal has been addressed with these basic techniques for a long time, the approach we advance here offers a significant advancement from the *status quo*. While visual inspection of EEG is still the normative procedure in neurology, the clear potential of computer-based assessments spawned a more quantitative approach over a generation ago.

Quantitative EEG (QEEG) summarizes an approach to EEG assessment on a few minutes of artifact-free data in a resting state, usually on a 19-channel clinical setup (John et al., 1988; Nuwer, 1997; Prichep, 2005; Coburn et al., 2006). The most common QEEG approaches use fast Fourier transforms (FFTs) to compute absolute and relative power at sites, hemispheric asymmetry of power, power ratios between pre-defined frequency bands, squared correlation of activity (“coherence”), phase lag times between electrode sites, or other related measures. These features are used to populate large-scale normative datasets to contrast with patient-specific databases, oftentimes statistically controlling for spurious variables like age. Finally, classification procedures like cross-validation and algorithms like discriminant analysis or neural networks may be applied in order to identify features that maximally discriminate patients from controls. This approach rests on the thesis that reliable statistical differentiation in the spatial representations of these multidimensional activities can differentiate a wide variety of psychiatric, congenital, and neurological disorders.

Notable successes include a Food and Drug Administration-approved prognostic biomarker for Attention-Deficit Hyperactivity Disorder in the ratio of theta to beta band power at the vertex electrode (Food and Drug Administration, 2013), and a candidate biomarker for acute traumatic brain injury (Thatcher et al., 1989; Naunheim et al., 2010; Prichep et al., 2014). While the validity and appropriate clinical utilization of these procedures remain highly debated (Arns et al., 2013; Saad et al., 2015; Gloss et al., 2016), resolution of these issues may be hamstrung by questionable premises underlying the general practice of QEEG (described below). We believe that the analytic approach motivated by PRED+CT will successfully address these problems and facilitate significant advancement in this field.

This QEEG approach has been highly controversial for a long time for a large number of reasons (Nuwer, 1997; Kaiser, 2000; Thatcher et al., 2003; Nuwer et al., 2005; Coburn et al., 2006). First, simple dissociation from statistical normality can easily be caused by the influence of spurious variables. Statistical dissociation has neither face-valid clinical implication nor clinical utility, as it doesn’t demonstrate that the differentiation will be faithfully reflected at the level of an individual (Amyot et al., 2015). Second, the QEEG approach relies primarily on resting activity, which has highly varied reliability across derived features (Nuwer et al., 2005), and lacks content and construct validity for assessing psychiatric and neurological disorders. Diagnostic criteria may even be the wrong target for associating with brain scans: identification of aberrant neural mechanisms underlying a disorder may be a more fruitful target than phenotypic characterization (Insel et al., 2010; Montague et al., 2012; Gillan and Daw, 2016). Third, the overleveraging of the FFT offers only a superficial decomposition of brain activities. Few studies have aimed to apply highly novel analytic techniques to derive maximally dissociating features of specific diseases (cf. Allen and Cohen, 2010; Lainscsek et al., 2013). Together, these issues reflect a fundamental failure to appreciate how EEG activities mechanistically reflect unique neural computations that may most parsimoniously define disease states.

In some cases, black-box techniques for pre-processing and quantification have been intertwined with privatization and commercialization. While commercialization is an important positive step toward clinical utility, it is sometimes necessarily closed source and has unfortunately been associated with overzealous advertisement and dubious claims (Nuwer et al., 2005; Coburn et al., 2006). In sum, while QEEG is a promising and selectively successful approach, current practice is highly limited. PRED+CT aims to motivate a common platform for methodological advancement, predicated on fully transparent databases and computational tools. Importantly, bigger data and increased algorithmic complexity are only part of the solution: we would like to highlight that success may depend on the ability to identify the right task to probe the aberrant mechanism underlying a specific disorder.

## PRED+CT

**FIGURE 1:**
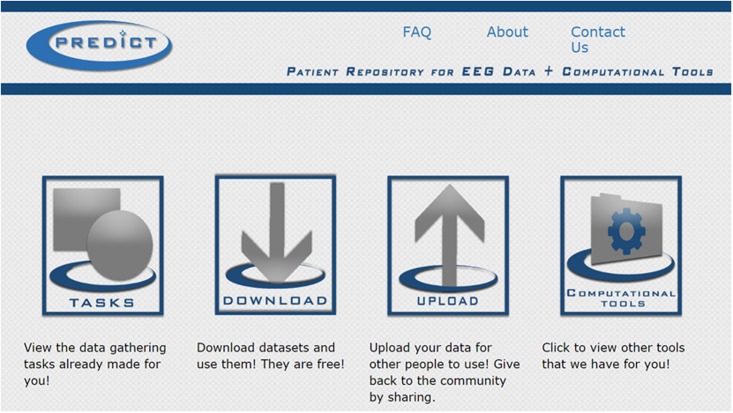
Screenshot of the PRED+CT home screen (www.predictsite.com).

The ultimate goal of this project is to host a multitude of tasks and patient datasets, facilitating large-scale data mining (**Figure 1**). We hope that successful completion of this aim will lead to development of novel EEG biomarkers with enhanced predictive power above and beyond phenotypic assessments for differentiating populations of neurological and psychiatric disorders.

### Tasks

To facilitate standardization across laboratories, we have developed software applications for oddball-type tasks (Sutton et al., 1965) and an Eriksen flankers task (Eriksen and Eriksen, 1974). These applications are coded in the Java programming language and work on Windows, but can also be run on other platforms through a Windows emulator. Each application allows the user to input a range of configurations to comprehensively cover parameter variations (inter-trial interval, visual or auditory modality, error feedback, instructions, etc.). A user can save a configuration file with these parameters, as well as any type of response requirement. This latter feature facilitates the creation of a variety of passive and active tasks, including go/no-go and vigilance tasks. By providing these programs, we hope to encourage smaller site-specific patient studies to include an additional short assessment to their protocols for the purpose of open-source data sharing.

If popular, we hope to include a multitude of task types in the future, such as reward gambling, stop-signal, basic language, and motor tasks, etc. Some existing task batteries for EEG assessment capitalize on simultaneous acquisition of a large number of EEG events/ERP components (Kappenman and Luck, 2012; Kieffaber et al., 2016; Nair et al., 2016); this may be a promising direction for future expansion.

### Upload and Download

An upload tab facilitates user requests for contributing data to PRED+CT. A download tab contains study information (**Table 2**) and will be fully open (no log in or request required). All data will be hosted in Matlab readable format (.mat or .set files, which are interchangeable) for a few reasons. Matlab is the current most common platform for academic EEG research, and the popular EEGLab suite (Delorme and Makeig, 2004) will be utilized as a common data structure. Many native file types contain information that could be a threat to confidentiality, and EEGLab import tools strip many of these markers. Matlab files are easily imported into other (open-source) programs like Octave, Python, and R, so this common structure should not be limiting in any way.

**Table 2:** Information required for datasets (EEG files) and tools (computer programs for analysis) to be contributed to PRED+CT.

Necessary information: datasets and tools	Necessary information: datasets only	Useful information: datasets only
Project name	Patient group/no.	Symptom scores
Lead investigator	Controls?/no.	Extended demographics
Funding (if applicable)	EEG system	Neuropsych scores
Publication link (if applicable)	Number of electrodes	Stimulus presentation files
Task		.mat file used for import
Brief description		
	
Email^†^	**Necessary information: dataset upload**	**Useful information: tools only**
	
	EEG data files	README-type description
	Age and sex of each participant	Example dataset
	Description of trigger types and any pre-processing performed	
	If not discernable from raw dataset: sampling rate, reference, electrode labels	


*^†^Email will only used for communication with PRED+CT administrators and will not be made public.*

### Confidentiality

The most likely threats to personal health information in candidate PRED+CT database entries are names or initials, locations, and dates. The user is required to ensure that none of these remain in the subject identifier or in the EEG metadata: for example, BrainVision.vhdr files contain times and .vmrk files contain a date stamp. EEGLab import to Matlab strips the data of such possible hidden threats to confidentiality. PRED+CT administrators will perform a double check on potential threats to confidentiality and can assist with the translation from native formats into .mat files. Most institutional review boards (IRBs) do not consider data sharing itself to fall under the definition of “human subjects research,” but interested users should request a determination from their IRB. Including such language in the informed consent is the best way to ensure that ethical issues are proactively well-managed.

### Data Organization

It is highly recommended that users include data in its raw form prior to any pre-processing; however, this may be infeasible in some cases. In many instances, important recording information is embedded in the native data format (sampling rate, reference, electrode labels) and is translated directly into the EEG data structure. If this information is missing (i.e., data are from a clinical system) then the user should include a readme file with this information.

For data other than rest, is important that users can understand what trigger types (TTL pulses) are used to represent each type of event. At minimum, a comprehensive list of trigger types needs to be included, and we highly recommend that users include the stimulus presentation script (i.e., Eprime run file, Matlab Psychtoolbox files, etc.). Ideally, the task was designed so that behavioral responses can be recoverable from the triggers; if not then upload of separate behavioral logs is encouraged. While there are common neuroinformatics structures that can facilitate sophisticated data organization schemes across studies (Teeters et al., 2008; Landis et al., 2016; Plis et al., 2016; Wiener et al., 2016), we opted for a more simplified approach in PRED+CT based on simple and well-documented descriptions of idiosyncratic TTL triggers.

### Computational Tools

Pattern classifiers use cross-validation or bootstrap approaches where the whole dataset is partitioned into non-overlapping training and test sets. The classifiers involve optimizing information theoretic and multidimensional metrics to generate models based on signal shapes and do not over-fit the data as traditional predictive models do (Parra et al., 2005; Pereira et al., 2009; Lemm et al., 2011). Such pattern-based classification is thus necessary to generalize predictive models for diagnostic subtyping and recovery trajectory to other groups (aka biomarkers).

There are a number of tutorials for general brain science classification (Pereira et al., 2009; Lemm et al., 2011), EEG-specific tutorials (Parra et al., 2005; Dyrholm and Parra, 2006; Schirrmeister et al., 2017), and open-source sets of analytic tools (Detre et al., 2006; Hanke et al., 2009). Our goal is not to replicate these resources, but to provide a repository for computational approaches that can bolster feature selection or patient classification, particularly on existing datasets in the archive. While we envision hosting a multitude of scripts, there is no reason that entries on this page couldn’t link to external resources (i.e., github or NITRC).

### Intellectual Property and Credit

Unless otherwise noted, this database and its contents are made available under the Public Domain Dedication and License v1.0 whose full text can be found at: https://opendatacommons.org/licenses/pddl/1.0/. We hope that all users will follow the ODC Attribution/Share-Alike Community Norms, including the expectation that there will be no attempt to de-anonymize any data.

### Example

**FIGURE 2:**
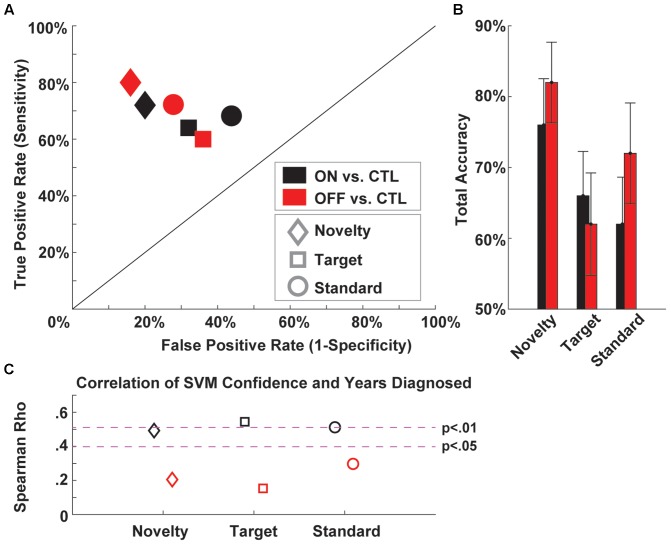
Example support vector machine (SVM) classification of Parkinson’s patients ON and OFF medication vs. well-matched controls based on three-auditory oddball task conditions. **(A)** A receiver operating characteristic plot shows the true vs. false positive rates of PD vs. CTL discrimination for each medication and task condition. **(B)** Total accuracy (average of sensitivity and specificity) for each condition. **(C)** Correlation of SVM confidence and years diagnosed for each condition. EEG data are available under Downloads (accession no.: d001) and Matlab scripts are available under Computational Tools (accession no.: t001).

**Figure 2** shows an example of the type of outcome we hope to cultivate with PRED+CT. This figure shows a receiver operating characteristic plot detailing classification of Parkinson’s patients on and off medication vs. well-matched controls based on three-auditory oddball task conditions (Cavanagh et al., under review). That report details the reasons why aberrant orienting to novelty is mechanistically interesting in Parkinson’s disease, and why EEG is uniquely well-suited to assess to biomarker potential of the associated neural response. These raw data and scripts are available on the PRED+CT website (accession nos.: d001 and t001). Since this task is very brief and very easy for patients to perform, we encourage other groups to contribute similar datasets to examine the generalizability of this phenomenon.

### Updates

Interested users can follow @PREDiCT_Admin or #PREDiCT + #UNM on Twitter for updates, including new dataset and tool contributions.

## Future Challenges to Surmount

### Challenges in Data Processing

While data sharing is “good,” a prevalent challenge is to share high-quality usable data (Kennedy, 2004). By archiving EEG and metadata in a common EEGLab structure, we can fulfill these criteria. Substantive hardware and software advancements over time are unlikely to change basic aspects of EEG data. Numerous algorithms exist to assist in pre-processing EEG data (Delorme and Makeig, 2004; Nolan et al., 2010; Oostenveld et al., 2011; Bigdely-Shamlo et al., 2015; Chaumon et al., 2015). In the Computational Tools section, we have provided our Algorithmic Pre-Processing Line for EEG (APPLE.m; accession no.: t002), which leverages a combination of FASTER (Nolan et al., 2010), ADJUST (Mognon et al., 2011), EEGLab, and custom algorithms for automatically interpolating bad channels, removing bad epochs, and identifying the most likely independent component associated with eye blinks.

Electroencephalography datasets come in a variety of reference schemes, topographical layouts, and sampling rates, complicating integration. However, these are addressable problems. Use of the average reference and relative measurements (decibel, percent change, relative power) facilitate common analytic space. EEG data tends to be oversampled, so down-sampling to a common lowest denominator is a viable option. As pattern classifiers leverage any difference between training sets, it will be critical to ensure that spurious differences between combined datasets do not interfere with the aim of classifying patients from controls. Having an equal number of patients and well-matched controls in each dataset is a good first step for experimental control over this issue, but additional steps like controlled randomization of training and testing sets may be required to control for dataset-specific biases.

Once pre-processed, EEG data offer a rather simple data structure that is accessible by non-experts. A two-dimensional matrix of channels ^∗^ time can be easily restructured to include a third dimension based on discrete events (i.e., the EEG.data field of EEGlab), and no special software, opaque statistical constraints, advanced processing, or other complicated considerations are strictly necessary for interpretation. We hope this increases the appeal to computer and data scientists, who should be able to manage EEG data as an input variable with very minimal special training.

### Challenges in Prediction

A well-known adage in machine learning is that achieving 80% classification accuracy is easy, and closing the gap toward 100% accuracy will take between a few years and eternity. We think that PRED+CT can assist with strategies for (partially) closing this gap, which we detail in order of their intuitiveness. The most straightforward solution to boost generalizability is to utilize larger training sets (i.e., more EEG data), which is the primary purpose of the site. Another immediately apparent solution is to leverage algorithmic advancements. In isolation it is hard to know how revolutionary different classification procedures will be, but open communication may help set standards and constraints on parameter selection which otherwise act as a hidden threat to generalizability. It is important to note that theoretical validation requires the ability to interpret which of the input features led to successful classification (cf. Steele et al., 2014; Cavanagh and Castellanos, 2016; Doshi-Velez and Kim, 2017).

We submit that the best way to achieve these overarching goals will involve taking advantage of the EEG feature(s) reflecting the neural computations that maximally discriminate groups. While resting activities may be the optimal solution for some patient groups, specifically designed active tasks are likely necessary to elicit the requisite brain responses that characterize the nature of the departure from statistical normality. Error signaling in anxiety has already been described above as a defining neural computation related to the etiology of the disorder, but other candidate responses have been advanced for other disorders, including diminished reward signals for major depression (Proudfit, 2015), a broken target-updating P3b in schizophrenia (Ford, 1999), a reduced novelty orienting P3a in Parkinson’s (Solís-Vivanco et al., 2015), and reduced brainstem evoked responses in acute traumatic brain injury (Kraus et al., 2016) to name just a few.

Finally, a less intuitive recipe for success may be to simply ask more specific questions. More constrained hypotheses can help to collapse insurmountable prior probabilities into the realm of plausibility. Consider that the base rate for any specific neurological or psychiatric disease is low enough to dismiss the plausibility of a new EEG-based diagnostic test with viable sensitivity and specificity. Yet if a patient is already being treated for a disease, this obviates some base rate problems. For example, instead of trying to develop a fast and easy brain scan to identify if someone has major depressive disorder, it is more plausible to ask if a diminished brain response to reward can help guide treatment options to address melancholic vs. atypical features of depression.

### Challenges in Diagnostics

To be medically useful, a test must have positive prognostic value above and beyond current *status quo*, or reduce time, cost, or uncertainty (Nuwer et al., 2005). While brain-based diagnostics may achieve impressive sensitivity, they are often associated with high false positives (low specificity), which is a particular deterrent to clinical use for differential diagnosis (Nuwer et al., 2005). These challenges are addressable, especially since direct clinical application is not a necessary outcome of brain-based patient classification.

High sensitivity in the context of low specificity may nevertheless have important clinical utility for rapidly assessing the potential presence of diagnostic complications (Hanley et al., 2013; Ayaz et al., 2015), quantifying the degree of injury severity (Thatcher et al., 2001), or for tracking differences in disease progression in treatment studies. Identification of the maximally discriminable neural computation that defines a patient group has additional translational utility: for instance it could be used as concurrent validation of a novel imaging or biomarker measure. In sum, reliable novel findings are important successes even if they do not lead to direct clinical translation.

## Conclusion

The genesis of PRED+CT was motivated by an understanding of the strength of EEG measurements and methods, but equally matched frustration in the logistical constraints of advancing beyond small-scale validation studies. EEG is a uniquely powerful measure of canonical neural operations, and machine learning has already led to profound social advancements. Surely we should have some firm answers to important clinical neuroscience questions by now. Yet single laboratory contributions to clinical science remain slow, expensive, time-consuming, and oftentimes led to beautiful but neglected datasets as interests and energies are applied to new funding opportunities. Only through collective action and full transparency can we hope to realize the utility of EEG-derived features of underlying neural computations for clinical neuroscience research.

## Author Contributions

JC and AM designed the project. AN and CW programed the site and tools. JC wrote the first draft.

## Conflict of Interest Statement

The authors declare that the research was conducted in the absence of any commercial or financial relationships that could be construed as a potential conflict of interest.

## Footnotes

1 www.predictsite.com
